# Micro‐CT imaging of *Onchocerca* infection of *Simulium damnosum s.l*. blackflies and comparison of the peritrophic membrane thickness of forest and savannah flies

**DOI:** 10.1111/mve.12509

**Published:** 2021-01-21

**Authors:** M. J. R. Hall, D. Martín‐Vega, B. Clark, D. Ghosh, M. Rogers, D. Pigoli, F. B. D. Veriegh, A. Tetteh‐Kumah, M. Y. Osei‐Atweneboana, R. A. Cheke

**Affiliations:** ^1^ Departments of Life Sciences and Core Research Laboratories Natural History Museum London UK; ^2^ Departamento de Ciencias de la Vida (Unidad Docente de Zoología) Universidad de Alcalá Alcalá de Henares (Madrid) Spain; ^3^ Nutrition and Clinical Services Division International Centre for Diarrhoeal Disease Research, Bangladesh (icddr,b) Dhaka Bangladesh; ^4^ Department of Disease Control London School of Hygiene and Tropical Medicine London UK; ^5^ Department of Mathematics King's College London London UK; ^6^ Council for Scientific and Industrial Research Water Research Institute Accra Ghana; ^7^ Agriculture, Health and Environment Department, Natural Resources Institute University of Greenwich Medway Campus Chatham Maritime Kent UK

**Keywords:** infection, micro‐CT, *Onchocerca*, peritrophic membrane, *Simulium*

## Abstract

Onchocerciasis is a neglected tropical disease (NTD) caused by *Onchocerca* Diesing 1841 (Spirurida: Onchocercidae) nematodes transmitted by blackflies. It is associated with poverty and imposes a significant health, welfare and economic burden on many tropical countries. Current methods to visualize infections within the vectors rely on invasive methods. However, using micro‐computed tomography techniques, without interference from physical tissue manipulation, we visualized in three dimensions for the first time an L1 larva of an *Onchocerca* species within the thoracic musculature of a blackfly, *Simulium damnosum s.l*. Theobald 1903 (Diptera: Simuliidae), naturally infected in Ghana. The possibility that thicker peritrophic membranes in savannah flies could account for their lower parasite loads was not supported, but there were limits to our analysis. While there were no statistically significant differences between the mean thicknesses of the peritrophic membranes, in the anterior, dorsal and ventral regions, of forest and savannah blackflies killed 34–48 min after a blood‐meal, the thickness of the peritrophic membrane in the posterior region could not be measured. Micro‐computed tomography has the potential to provide novel information on many other parasite/vector systems and impactful images for public engagement in health education.

## Graphical abstract

Micro‐computed tomography was used to visualize in 3D an L1 larva of an *Onchocerca* species within the thoracic musculature of a blackfly, *Simulium damnosum s.l*., naturally infected in Ghana.
There were no statistically significant differences between the mean thicknesses of the peritrophic membranes of forest and savannah blackflies killed 34–48 min after a blood‐meal.
Micro‐computed tomography has the potential to provide novel information on many other parasite/vector systems and impactful images for public engagement in health education.

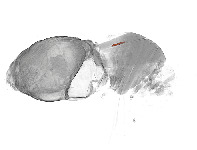

## Introduction

Onchocerciasis is a neglected tropical disease (NTD) caused by *Onchocerca volvulus* (Leuckart 1893) (Spirurida: Onchocercidae) nematodes that are transmitted by blackflies, *Simulium* Latreille 1802 species (Diptera: Simuliidae). It is associated with poverty and so‐called “resource poor” settings, where it can seriously reduce household productivity (Ibe *et al*., 2015) and it imposes a significant health, welfare and economic burden on many tropical countries. The main focus of onchocerciasis burden remains in Africa (Colebunders *et al*., 2018a) where a 2017 study estimated that 99% of worldwide cases occurred, with almost 21 million people infected in 31 countries (CDC, 2020). Following treatments with the microfilarial drug ivermectin in the Americas, the disease there is now restricted to a focus straddling the border between Brazil and Venezuela (CDC, 2020; Gustavsen *et al*., 2011). Onchocerciasis not only causes blindness and skin disease, but there is growing evidence that it is associated with epilepsy and nodding syndrome (Colebunders *et al*., 2018b; Vinkeles Melchers *et al*., 2018).

In research on the infection of vectors with disease agents that cause NTDs, there is scope for visualization techniques to improve the fundamental understanding of parasite‐vector interactions, the basis of vectorial competence and transmission. Precise spatio‐temporal information on the progress of an infection can tell us much about the interactions required for successful colonization and transmission. However, visualization of these interactions within the vectors, including *Onchocerca* within *Simulium*, has hitherto proven difficult, relying on invasive dissection methods (e.g. Bain, 1969; Trpis *et al*., 2001). Micro‐computed tomography, micro‐CT, has proven to be a powerful non‐invasive tool for visualizing the internal morphology of insects, such as the intra‐puparial development and metamorphosis within the puparium of blow flies (Diptera: Calliphoridae) and bot flies (Diptera: Oestridae) (Hall & Martín‐Vega, 2019; Martín‐Vega *et al*., 2017, 2020). It has also recently been seen as an emerging opportunity in parasite imaging, used to reveal parasitization of mice with the mouse whipworm, *Trichuris muris* (Schrank 1788) (Trichocephalida: Trichuridae), a nematode (O'Sullivan *et al*., 2018), as well as of ants infected with the lancet liver‐fluke, *Dicrocoelium dendriticum* (Rudolphi 1819) (Plagiorchiida: Dicrocoeliidae) (Martín‐Vega *et al*., 2018).

In onchocerciasis studies it remains unclear why some species are good vectors and others are inefficient at or incapable of transmission (Adler *et al*., 2010; Cheke & Garms, 2013). The peritrophic membrane (PM: note that Terra *et al*., 2019, recommend avoiding the use of the term peritrophic matrix) is one potential anatomical structure in the blackfly that could have a role to play in infection post blood‐meal as the ingested microfilariae have to pass through it towards the ecto‐peritrophic space and beyond to establish an infection (Bain & Philippon, 1970; Basáñez *et al*., 2009). The rate of hardening of the PM and its final thickness are considered likely to influence the vector capacity of the blackfly (Ramos *et al*., 1994). The membrane forms within 30 s after a blood meal, enveloping the ingested blood and often trapping ingested microfilariae of *Onchocerca*, which die and are digested within it (Bain & Philippon, 1970; Lewis, 1953). One hypothesis is that the PM is thicker and better sealed in savannah than in forest members of the *S. damnosum* complex, such that the latter can support more *Onchocerca* larvae (Bain *et al*., 1976; Philippon, 1977). Eichner *et al*. (1991) showed that for Cameroonian species of *Simulium*, savannah flies supported the development of forest *O. volvulus* better than forest flies after infection by intrathoracic injection of microfilariae. This was the reverse of the result after natural *per os* infections. Therefore, they concluded that the PM was the main barrier against larval development after ingestion of infected blood by the Cameroonian vectors. Rose *et al*. (2020) used a number of microscopy techniques not only to visualize trypanosome infection within the region of the proventriculus of tsetse flies, *Glossina morsitans morsitans* Westwood 1851 (Diptera: Glossinidae), but also to measure the thickness of the PM. However, their technique, like that used to reveal *Onchocerca* within the blackfly host, required dissection of the flies.

The objective of our study was to determine if micro‐CT could be used for non‐invasive studies of the infection of blackflies with *Onchocerca*, without interference from direct, physical tissue manipulation (tissues were only fixed and stained), specifically to visualize *Onchocerca* infection and measure PM thickness in whole flies, without dissection. We report here the visualization in three dimensions (3D) for the first time of an L1 larva of an *Onchocerca* species within the thoracic musculature of a blackfly, *S. damnosum s.l*., naturally infected in Ghana.

## Materials and methods

### Specimen collection

Blackfly pupae were collected from Boti Falls on the Pawnpawn river in Ghana (06° 10′ N, 00° 11′ W) on 6 July 2019. This site is a well‐known breeding site for the forest cytospecies *Simulium squamosum* (Enderlain 1921) cytoform “E”. Adults were reared from the pupae. Only female flies were kept, each in separate tubes. Similar methods were used to obtain adult female *S. sanctipauli* Vajime & Dunbar 1975, “Pra” cytoform from the Pra River at Sekyere‐Hemang (05° 11′ N, 01° 35′ W), another forest site, on 11 July 2019. Savannah flies (*S. damnosum* Theobald 1903 *s.str*./*S. sirbanum* Vajime & Dunbar 1975) were obtained from Tainso (=Nipanekro, 08° 06′ N, 02° 06′ W) on the River Tain, a tributary of the Black Volta river. The adult flies were kept cool using a portable refrigerator (Dometic Tropicool model TCX14).

Initial attempts to feed some of the flies on cattle, as originally planned, were mostly unsuccessful. After identifying cattle with obvious nodules indicative of infections with *Onchocerca ochengi* Bwangomoi 1969, only three flies were fed on a cow at Agborlekame (08° 14' N, 02° 12′ W), despite extensive efforts, including placing flies within a muslin sleeve on a cow's hind leg. Attempts with other cattle at Tainso failed as the selected cow, with obvious nodules, could not be restrained for long enough.

To obtain blood‐fed flies, samples were fed on human volunteers at Agborlekame and Tainso. The volunteers were known onchocerciasis patients, identified as such during ongoing studies on use of ivermectin for onchocerciasis control (by M. Osei‐Atweneboana). In some cases, opportunistic wild flies fed on and were collected from other volunteers, after which these volunteers were given ivermectin to eliminate the possibility of the transmission of infection.

To obtain samples for examining and measuring the PM, samples were killed 25–161 min after blood‐feeding. In the hope of obtaining flies infected with larvae of *O. volvulus*, other samples were maintained alive in tubes with sugared water within the portable refrigerator for various times up to 10 320 min (7.2 days) after the blood‐meals, since earlier studies have shown that flies six or more days old were capable of harbouring infective L3 larvae (Duke, 1968).

### Staining protocols

To increase X‐ray absorption of low‐density tissues and enhance overall contrast, blackflies were stained for one week by immersion in a solution of 1% phosphotungstic acid (PTA) in 80% ethanol, with continual swirling on a laboratory mixer and a change to fresh staining solution mid‐period. Fly legs were removed prior to staining to enhance stain penetration. The PTA bound permanently to the fly tissues and so material could be held for a lengthy period before scanning without loss of stain (Metscher, 2009).

### Micro‐CT studies

Twenty‐five blackflies were scanned, 10 to check for *Onchocerca* larval infestation (at least 8865 min post‐feed, i.e. >6 days) and 15 to measure the PM soon after feeding (twelve at 34–48 min post‐feed, three at 157–161 min post‐feed).

For scanning, each specimen was placed in 80% ethanol in a 0.2 mL micro‐centrifuge tube and scanned in a Zeiss Versa 520 system (Carl Zeiss Microscopy GmbH, Jena, Germany) with 4x optical magnification, with exposure set to 12 s, current to 83 μA and voltage to 60 kV. The resulting projections were reconstructed as tiff stacks with a voxel size of 0.9–1.3 μm^3^. Reconstructed data were imported into VG Studio Max 2.2 (Volume Graphics GmbH, Heidelberg, Germany), in which slice stacks were rendered, reoriented and visualized in the three principal planes (cross, horizontal and sagittal). The reconstructed data were also loaded into Avizo Version 2019.1 (Visualization Sciences Group, Bordeaux, France) for manual segmentation, volumetric measurement and 3D visualization. Volumetric measurement was performed using the Avizo's ‘Material statistics’ module. All uses of ‘segmentation’ and ‘segmented’ in this study refer to the manual or automated process of assigning a label to all voxels that correspond to a certain structure, thereby enabling that structure to be separated from the voxels of other tissues or of the background (Rovaris *et al*., 2018). For example, in this study, the *Onchocerca* larva was segmented by labelling all the voxels that made up its structure, thereby enabling it to be visualized in 3D and its volume to be measured by, respectively, selecting and counting the labelled voxels.

### Measurement of thickness of peritrophic membrane

Of the 15 blackflies scanned to compare the thickness of the PMs of forest and savannah flies, 10 flies killed in the same period, 34–48 min post‐feed, were measured, five forest and five savannah flies. The mean time after completion of a bloodmeal that forest and savannah flies were killed was 41.2 and 44.6 min, respectively. These blackflies were the first five of each fly group examined with image quality that enabled all the measurements detailed below to be made. Other flies were scanned, but it was not possible to unambiguously discriminate the PM at all sites chosen for measurement and so these flies were rejected.

On the medial sagittal 2D virtual section of each specimen that was used in the analysis, three measurements were made of the PM thickness as evenly distributed as possible along each of the anterior, dorsal and ventral regions (i.e., measurements at nine PM locations per section per specimen). The PM at the posterior region was very thin and hardly discernible, so this region was not considered for measurements. The same procedure was repeated on two other sagittal virtual sections on each side of the body, scrolling 30–35 sections laterally and then a further 30–35 sections for both right and left body sides. Hence, 45 measurements (at three anterior, three dorsal and three ventral locations on five sagittal virtual sections) of PM thickness were performed on each specimen. Measurements were performed using Avizo's ‘Measurement’ tool.

We carried out a statistical analysis of the PM thickness data by fitting an analysis of variance (ANOVA) model with repeated measurement, but allowing for different error variances for each PM region of each fly. This was done by fitting a mixed effects ANOVA model (Pinheiro & Bates, 2000) with PM region and fly as (nested) random effects using the R Statistical Software (R Core Team, 2020) and the nlme package (Pinheiro *et al*., 2020).

## Results

### Visualization of bloodmeal

**Fig. 1:**
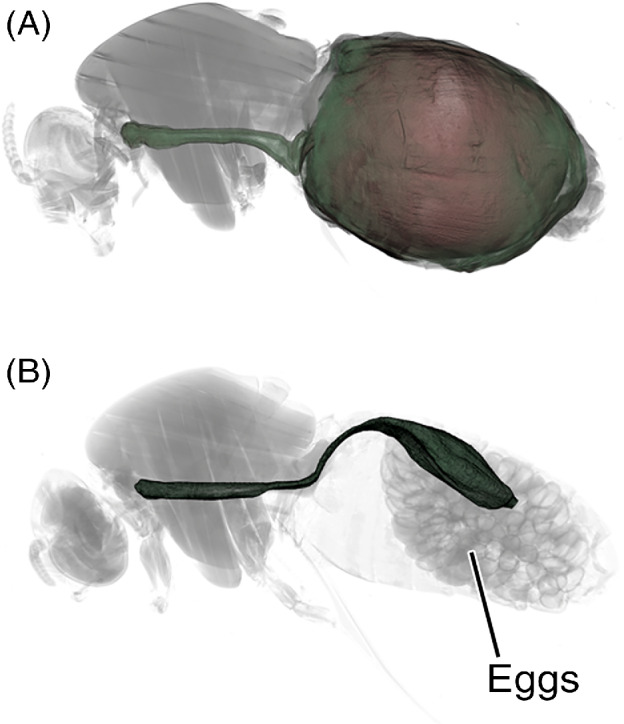
False‐coloured 3D images of blackflies killed either 40 min (A, forest specimen) or 10 320 min (B, 7.2 days, savannah specimen) after completion of a blood meal. Note developing eggs in B.

The specimens collected ranged from 25 to >10 000 min post‐feed and so presented an opportunity to visualize the dramatic change in the gut volume as the bloodmeal was digested (Fig. 1).

### *Visualization of infection with* Onchocerca

**Fig. 2:**
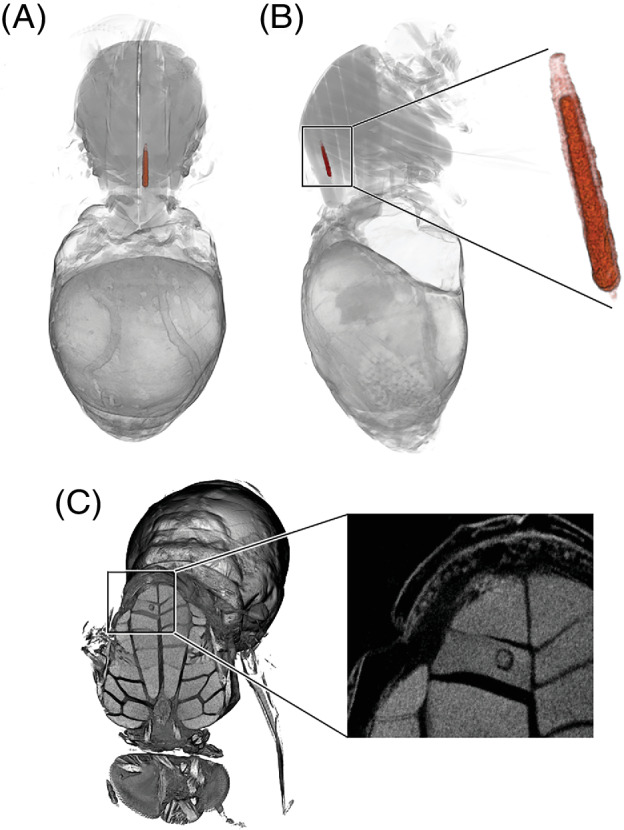
Dorsal (A) and lateral (B) false‐coloured 3D images of a larva of *Onchocerca* in the flight muscle of *Simulium damnosum s.l*. (savannah specimen), with a higher resolution image in B just of the larva (dark red) within the intramuscular space (light red). A virtual 3D cross‐section through the thorax of the fly reveals the larva within the flight muscle block (C), which is shown in more detail in a higher resolution virtual 2D cross section. The larva had a length of 218.8 μm, a width of 23–26 μm and volume of 65 898 μm^3^, occupying 44.9% of the intramuscular space.

**Fig. 3:**
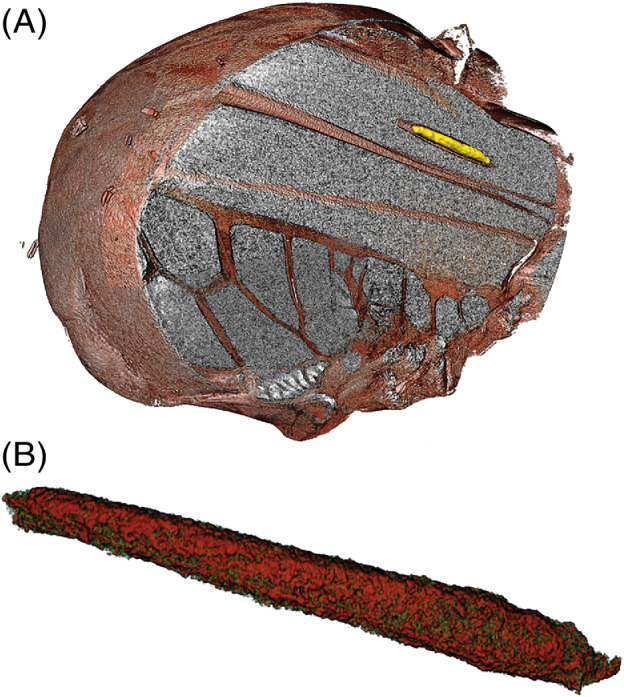
(A) False‐coloured 3D illustration of a larva of Onchocerca sp. within an intramuscular space in the flight muscle of *Simulium damnosum s.l*. (savannah specimen) with the thorax cut away to reveal the larva (yellow, with surface smoothed by Avizo software). (B) False‐coloured illustration of the same larva virtually dissected (segmented) away from the fly tissues to enable 2D measurement of length and width.

Just one of the scanned blackflies (1/25 = 4%), an opportunistic wild feeder, was found to be infected. This came from the group that had recently fed, whereas none of the ten flies that had fed more than six days before killing were infected. The infected fly had a single first stage (L1) *Onchocerca* larva, of the so‐called ‘sausage’ (‘saucisse’) shape (Philippon, 1978), located in the indirect flight muscles (Figs 2 and 3). The larva was segmented in Avizo and visualized in detail, measuring its volume and that of the intramuscular space it occupied. The larva had a length of 218.8 μm, a width of 23–26 μm and volume of 65 898 μm^3^, occupying 44.9% of the intramuscular space that had been formed.

### Thickness of peritrophic membrane

Discrimination of the PM was not always easy due to similar X‐ray opacity of adjacent tissues, resulting in poor contrast and poor boundary definition. Therefore, measurements were only included where the PM thickness was unambiguous. Despite these difficulties, no differences were detected between the measurements made by individual investigators and so they were all pooled and analysed together.

**Fig. 4:**
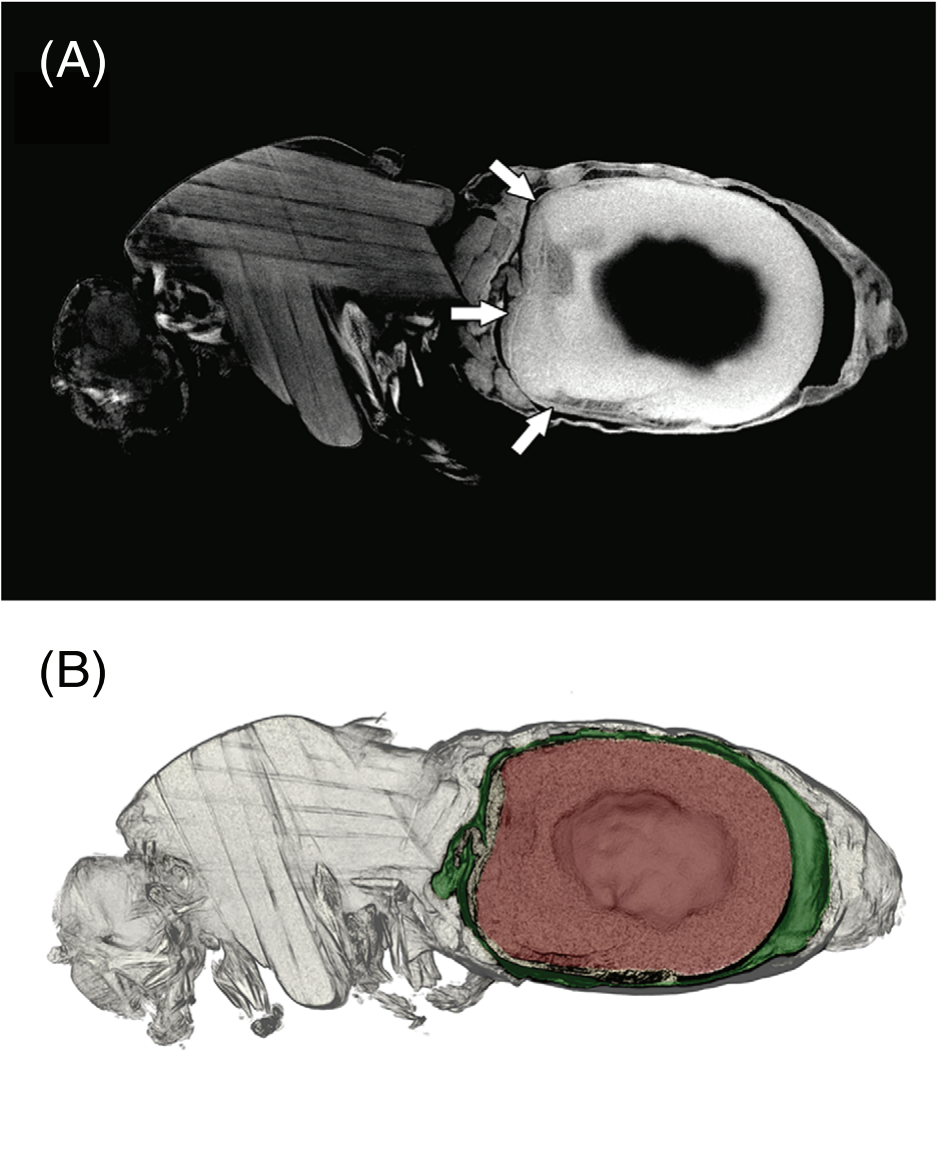
(A) Measurement of PM thickness (arrowed at dorsal, anterior and ventral positions) from mid‐sagittal 2D virtual section of *Simulium damnosum s.l*. (forest specimen). (B) Same specimen with false colouring of a 3D virtual section at the same position as in A, showing midgut (green), bloodmeal (red) with peritrophic membrane left as the grey tissue between the coloured layers.

Micro‐CT scans showed the PM in both forest and savannah blackflies preserved soon after feeding (Fig. 4). The bloodmeals always appeared to be surrounded by the PM, certainly in the anterior and middle regions, but in the posterior region it was often difficult to discern in both groups of flies and so its thickness there was not measured.

**Fig. 5:**
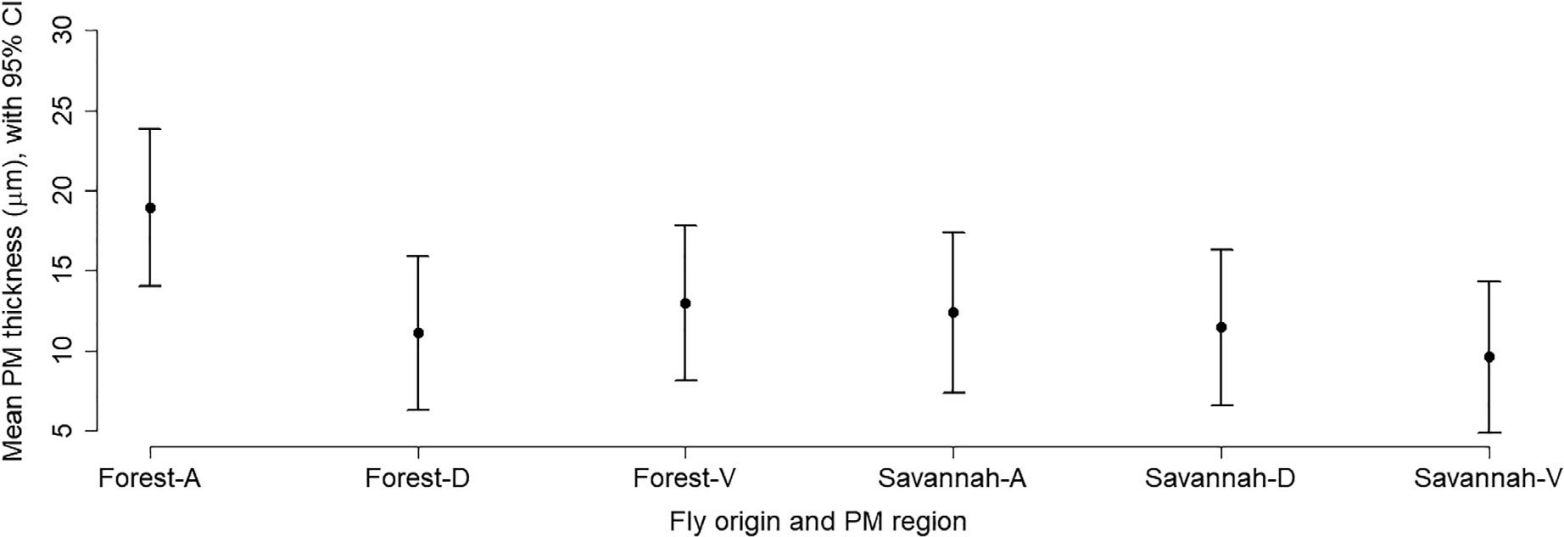
Plot of peritrophic membrane (PM) thickness (means ±95% CI, *n* = 5 flies per graph point) in three PM regions (A = anterior, D = dorsal and V = ventral PM) for blackflies of either forest or savannah origin, killed 34–48 min after a human blood‐meal.

The estimated mean PM thickness was slightly larger for forest flies than for savannah flies, but the difference was not statistically significant (estimated difference = 6.584 μm, standard error = 3.2877 μm, approximated df = 9, *P* value = 0.0762). There was indeed a large variability in the estimated error variance between PM regions and across flies. For example, the estimated standard deviation was larger than 2 μm for the measurements of the savannah fly T2 in the anterior region, while it was less than 0.5 μm in the dorsal region of savannah fly T10. Figure 5 shows the estimated means of the PM thickness for each PM region (anterior, dorsal or ventral) and for each fly origin (savannah or forest), along with 95% confidence intervals (approximated df = 15).

## Discussion

Previous studies have shown that adult *S. damnosum s.l*. that have fed on humans infected with *O. volvulus* have a potential infection rate of 80% or more (Philippon, 1977). The 4% infection rate that we recorded here was, therefore, very low, but this may have reflected both the small sample size and the effects of the community directed treatments with ivermectin (CDTI) conducted throughout the study zone, although the efficiency of these vary geographically within Ghana (Frempong *et al*., 2016). The fly in question was a wild fly of savannah origin, caught at Tainso.

Using non‐invasive micro‐CT techniques on the single infected fly that we did find, we were able to visualize an *Onchocerca* larva *in situ* within its host blackfly for the first time. However, we were unable to determine if the imaged larva was of *O. volvulus* or of the cattle infecting species *O. ochengi*. Morphological identification of larvae of *Onchocerca* species is possible but very difficult and so genetic discrimination is a more rapid and reliable method (Doyle *et al*., 2016). A 48‐h post‐infection first stage larva of *O. volvulus* dissected from *S*. *damnosum s.l*. was reported to have a length and width of 210 μm and 18 μm, respectively (Bain, 1969). At 96 h post‐infection a first stage larva had comparable dimensions of 260 μm and 40 μm (Bain, 1969). Trpis *et al*. (2001) measured first stage larvae of *O. volvulus* developing in *S. yahense* Vajime & Dunbar 1975 and recorded mean lengths of 226, 171, 243 and 269 μm, respectively, 24, 48, 72 and 96 h after feeding. Our larva was slightly larger than the 48‐h specimens of both studies, but it is not possible to say if it was in the shortening or lengthening phase noted by Trpis *et al*. (2001) before and after 48 h. The fly was killed just 45 min after feeding and, therefore, this larva was clearly acquired from an earlier feed. There was just a single larva present in the scanned fly, but this is typical of savannah blackfly infections, which in most cases have from one to three infective larvae (L3) (Davies & Crosskey, 1991), with means of 1.9 L3 per infective fly, whereas mean numbers of L3s per infected forest fly range from 4.2 to 6.6 depending on cytoform (Cheke & Garms, 2013).

The PM of the scanned flies was sufficiently clear in those flies included in the analysis that its thickness could be measured. In some specimens, individual layers could be discerned and an anterior extension from the abdominal midgut into the non‐extended, thoracic region of the midgut. However, traditional light microscopy following histological staining and transmission electron microscopy studies deliver better resolution of the PM, albeit with invasive and destructive consequences for the specimens (e.g. Ramos *et al*., 1994; Reid & Lehane, 1984; Rose *et al*., 2020; Sádlová & Volf, 2009).

The mean thickness of the PM measured here, in flies killed 34–48 min after completion of feeding, was mostly in the range of 10–15 μm (Fig. 5). This is slightly thicker than the means of 2.6, 4.3, 8.1 and 9.3 μm, respectively, for the PM measured one hour after feeding of the temperate blackfly species *Simulium vittatum* Zetterstedt 1838 (1.5 h after feeding; Ramos *et al*., 1994), *S. equinum* (Linnaeus 1758)*, S. lineatum* (Meigen 1804) and *S. ornatum* Meigen 1818 (Reid & Lehane, 1984). Like us, Reid & Lehane (1984) observed great variation in the thickness of the PM, in all species they studied and at all times, with some species showing distinct layers and some none. As the PM acts as a barrier to infection of the fly with *Onchocerca*, this potential great variation in its thickness probably explains the great variation in fly infection efficiency, with Duke & Lewis (1964) recording from 0‐96% of ingested microfilaria escaping the membrane within 6‐10 h, depending on the fly dissected, a mean proportion of 44%. Of microfilariae that did escape the PM, it was estimated that 91% developed to infective larvae.

Our results do not support the hypothesis that forest blackflies have a thinner PM, which would allow them to develop more *Onchocerca* L3 larvae than savannah blackflies. However, our sample sizes were small and we were unable to measure the thickness of the PM of either group of flies in the posterior region. Therefore, not knowing where the larvae pass through the PM, we cannot rule out the possibility that differences between savannah and forest flies in that posterior region might contribute to a difference in susceptibility to *Onchocerca* infection. It would clearly be worthwhile repeating these studies on blackflies that were collected sooner after feeding than our flies, when the PM would be thinner (Reid & Lehane, 1984), ideally within the first 30 min of feeding when most microfilariae that do escape the PM have been recorded to escape (Laurence, 1966). Some microfilariae can even be detected within the thorax within 30 min of completion of feeding (Basáñez *et al*., 2009). The original hypothesis that we were testing is probably overly simplistic, because Eichner *et al*. (1991, referring to Duke *et al*., 1966) reported that, “In Cameroon, microfilariae of the forest strain develop much better in blackflies from the forest than in those from the [Sudan‐]savanna and *vice‐versa*”. Nevertheless, it remains possible that more larvae can penetrate the PM in forest flies if there are differences in the speeds of the membranes' development or if those of the forest flies do not get so well sealed, but our data suggest that alternative explanations for the observed differences in parasitism rates need to be sought.

Our initial studies have shown that micro‐CT investigations can complement other fundamental studies of the development of NTD disease agents within their vector hosts, thereby helping to highlight new avenues for tackling the disease in the vector. The specimen staining took one week but required no operator input. The image processing took in the order of 2–5 h per specimen depending on what was required. This is undoubtedly longer than a classic dissection, but different and unique sets of information (such as structure volumes) can be delivered. For onchocerciasis, it will be possible to follow progress of *Onchocerca* development within a series of infected flies, shedding light on its differential development within different vector species. Our measurement of the intramuscular space occupied by the larva demonstrates that it will also be possible to investigate the damage done to the blackfly tissues due to *Onchocerca* infection, both qualitatively and quantitatively. The damage caused by third stage larvae is especially profound (Trpis *et al*., 2001), with potential consequences for fly survival and, therefore, transmission.

In addition, health education has been identified as crucial in efforts to control or eliminate NTDs (Dejeux, 2001). Images produced by this study and future studies could have significant value in health education. In conclusion, although this initial study focused on blackflies, it demonstrates the potential for wider use of micro‐CT in the research and management of NTDs, providing novel information on many other parasite/vector systems and impactful images for public engagement.

## Ethical approval

The protocols used in this study were reviewed and approved by the Institutional Review Board of the Council for Scientific and Industrial Research (reference RPN 014/CSIR‐IRB/2019), Accra, Ghana.

## Data Availability

The data that support the findings of this study are available from the corresponding author upon reasonable request.

## References

[mve12509-bib-0001] Adler, P.H., Cheke, R.A. & Post, R.J. (2010) Evolution, epidemiology, and population genetics of black flies (Diptera: Simuliidae). Infection, Genetics and Evolution, 10, 846–865. 10.1016/j.meegid.2010.07.003. 20624485

[mve12509-bib-0002] Bain, O. (1969) Morphologie des stades larvaires d'*Onchocerca volvulus* chez *Simulium damnosum* et redescription de la microfilaire. Annales de Parasitologie Humaine et Comparée, 44, 69–81. 5392666

[mve12509-bib-0003] Bain, O. & Philippon, B. (1970) Mecanisme de la traverse de la paroi stomacale par les microfilaires chez *Anopheles stephensi* et *Simulium damnosum*. Mise en evidence d'un sejour des microfilaires dans l'epithelium digestif. Annales de Parasitologie Humaine et Comparée, 45, 295–320. 4395663

[mve12509-bib-0004] Bain, O., Philippon, B., Séchan, Y. & Cassone, J. (1976) Corrélation entre le nombre de microfilaires ingérées et l'épaisseur de la membrane péritrophique du vecteur dans l'onchocercose de savane africaine. Comptes Rendus Hebdomadaires des Séances de l'Académie des Sciences. Série D: Sciences Naturelles, 283, 391–392. 825287

[mve12509-bib-0005] Basáñez, M.‐G., Churcher, T.S. & Grillet, M.‐E. (2009) *Onchocerca‐Simulium* interactions and the population and evolutionary biology of *Onchocerca volvulus* . Advances in Parasitology, 68, 263–313. 10.1016/S0065-308X(08)00611-8. 19289198

[mve12509-bib-0006] CDC, Centers for Disease Control and Prevention (2020) Parasites – Onchocerciasis (also known as River Blindness). Onchocerciasis FAQs. https://www.cdc.gov/parasites/onchocerciasis/gen_info/faqs.html [accessed on 11 June 2020].

[mve12509-bib-0007] Cheke, R.A. & Garms, R. (2013) Indices of onchocerciasis transmission by different members of the *Simulium damnosum* complex conflict with the paradigm of forest and savanna parasite strains. Acta Tropica, 125, 42–53. 10.1016/j.actatropica.2012.09.002. 22995985

[mve12509-bib-0008] Colebunders, R., Basáñez, M.‐G., Siling, K. *et al*. (2018a) From river blindness control to elimination: bridge over troubled water. Infectious Diseases of Poverty, 7, 21. 10.1186/s40249-018-0406-7. 29587844 PMC5872540

[mve12509-bib-0009] Colebunders, R., Nelson Siewe, F.J. & Hotterbeekx, A. (2018b) Onchocerciasis‐associated epilepsy, an additional reason for strengthening onchocerciasis elimination programs. Trends in Parasitology, 34, 208–216. 10.1016/j.pt.2017.11.009. 29288080

[mve12509-bib-0010] Davies, J.B. & Crosskey, R.W. (1991) *Simulium* ‐ Vectors of Onchocerciasis, Vector Control Series Advanced Level Training and Information Guide, World Health Organisation, Division of Control of Tropical Diseases, WHO/VBC/91.992, 115 pp.

[mve12509-bib-0011] Dejeux, P. (2001) The increase in risk factors for leishmaniasis. Transactions of the Royal Society of Tropical Medicine and Hygiene, 95, 239–243. 11490989 10.1016/s0035-9203(01)90223-8

[mve12509-bib-0012] Doyle, S.R., Armoo, S., Renz, A., Taylor, M.J., Osei‐Atweneboana, M.Y. & Grant, W.N. (2016) Discrimination between *Onchocerca volvulus* and *O. ochengi* filarial larvae in *Simulium damnosum* (*s.l*.) and their distribution throughout central Ghana using a versatile high‐resolution speciation assay. Parasites and Vectors, 9, 536. 10.1186/s13071-016-1832-7. 27724959 PMC5057476

[mve12509-bib-0013] Duke, B.O.L. (1968) Studies on factors influencing the transmission of onchocerciasis. V. The stages of *Onchocerca voluvulus* in wild ‘forest’ *Simulium damnosum*, the fate of the parasites in the fly and the age‐distribution of the biting population. Annals of Tropical Medicine and Parasitology, 62, 107–116. 5679813

[mve12509-bib-0014] Duke, B.O.L. & Lewis, D.J. (1964) Studies on factors influencing the transmission of onchocerciasis. III. Observations on the effect of the peritrophic membrane in limiting the development of *Onchocerca volvulus* microfilariae in *Simulium damnosum* . Annals of Tropical Medicine and Parasitology, 56, 83–88. 14147669

[mve12509-bib-0015] Duke, B.O.L., Lewis, D.J. & Moore, P.J. (1966) *Onchocerca*‐*Simulium* complexes. I. Transmission of forest and Sudan‐savanna strains of *Onchocerca volvulus* from Cameroon by *Simulium damnosum* from various West African bioclimatic zones. Annals of Tropical Medicine and Parasitology, 60, 318–336. 5971132

[mve12509-bib-0016] Eichner, M., Renz, A., Wahl, G. & Enyong, P. (1991) Development of *Onchocerca volvulus* microfilariae injected into *Simulium* species from Cameroon. Medical and Veterinary Entomology, 5, 293–297. 1768922 10.1111/j.1365-2915.1991.tb00555.x

[mve12509-bib-0017] Frempong, K.K., Walker, M., Cheke, R.A. *et al*. (2016) Does increasing treatment frequency address sub‐optimal responses to ivermectin for the control and elimination of river blindness? Clinical Infectious Diseases, 62, 1338–1347. 27001801 10.1093/cid/ciw144 PMC4872292

[mve12509-bib-0018] Gustavsen, K., Hopkins, A. & Sauerbrey, M. (2011) Onchocerciasis in the Americas: from arrival to (near) elimination. Parasites and Vectors, 4, 205. 10.1186/1756-3305-4-205. 22024050 PMC3214172

[mve12509-bib-0019] Hall, M.J.R. & Martín‐Vega, D. (2019) Visualization of insect metamorphosis. Philosophical Transactions of the Royal Society B, 374, 20190071. 10.1098/rstb.2019.0071. PMC6711283 31438819

[mve12509-bib-0020] Ibe, O., Onwujekwe, O., Uzochukwu, B., Ajuba, M. & Okonkwo, P. (2015) Exploring consumer perceptions and economic burden of onchocerciasis on households in Enugu State, South‐East Nigeria. PLoS Neglected Tropical Diseases, 9, e0004231. 26618633 10.1371/journal.pntd.0004231 PMC4664248

[mve12509-bib-0021] Laurence, B.R. (1966) Intake and migration of the microfilariae of *Onchocerca volvulus* (Leuckart) in *Simulium damnosum* Theobald. Journal of Helminthology, 40, 337–342. 5962367 10.1017/s0022149x00020976

[mve12509-bib-0022] Lewis, D.J. (1953) *Simulium damnosum* and its relation to onchocerciasis in the Anglo‐Egyptian Sudan. Bulletin of Entomological Research, 43, 597–644. 10.1017/s0007485300030078 20287801

[mve12509-bib-0023] Martín‐Vega, D., Simonsen, T.J. & Hall, M.J.R. (2017) Looking into the puparium: Micro‐CT visualisation of the internal morphological changes during metamorphosis of the blow fly, *Calliphora vicina*, with the first quantitative analysis of organ development in cyclorrhaphous Diptera. Journal of Morphology, 278, 629–651. 28182298 10.1002/jmor.20660 PMC5412940

[mve12509-bib-0024] Martín‐Vega, D., Garbout, A., Ahmed, F. *et al*. (2018) 3D virtual histology at the host/parasite interface: visualisation of the master manipulator, *Dicrocoelium dendriticum*, in the brain of its ant host. Scientific Reports, 8, 8587. 10.1038/s41598-018-26977-2. 29872086 PMC5988677

[mve12509-bib-0025] Martín‐Vega, D., Clark, B., Ferrer, L.M., López‐Tamayo, S., Colwell, D.D. & Hall, M.J.R. (2020) Internal morphological changes during metamorphosis in the sheep nasal bot fly, *Oestrus ovis* . Medical and Veterinary Entomology, 34, 476–487. 10.1111/mve.12465. 32767606

[mve12509-bib-0026] Metscher, B.D. (2009) MicroCT for developmental biology: a versatile tool for high‐contrast 3D imaging at histological resolutions. Developmental Dynamics, 238, 632–640. 10.1002/dvdy.21857. 19235724

[mve12509-bib-0027] O'Sullivan, J.D.B., Behnsen, J., Starborg, T. *et al*. (2018) X‐ray micro‐computed tomography (μCT): an emerging opportunity in parasite imaging. Parasitology, 145, 848–854. 10.1017/S0031182017002074. 29179788 PMC6088774

[mve12509-bib-0028] Philippon, B. (1977) Etude de la transmission *d'Onchocerca volvulus* (Leuckart,1893) (Nematoda: Onchocercidae) par *Simulium damnosum* Theobald,1903 (Diptera:Simuliidae) en Afrique tropicale. Travaux et Documents de l'ORSTOM, Paris.

[mve12509-bib-0029] Philippon, B. (1978) L'onchocercose humaine en Afrique de l'Ouest. Initiations – Documentations Techniques No. 37, ORSTOM, Paris, 199 pp.

[mve12509-bib-0030] Pinheiro, J.C. & Bates, D.M. (2000) . Mixed‐Effects Models in S and S‐PLUS, p. 527 New York, USA: Springer‐Verlag.

[mve12509-bib-0031] Pinheiro, J., Bates, D., DebRoy, S., Sarkar, D. & R Core Team (2020) nlme: linear and nonlinear mixed effects models. R package version 3.1‐148. https://CRAN.R‐project.org/package=nlme

[mve12509-bib-0032] R Core Team (2020) R: A language and environment for statistical computing. R Foundation for Statistical Computing, Vienna, Austria. https://www.r‐project.org/.

[mve12509-bib-0033] Ramos, A., Mahowald, A. & Jacobs‐Lorena, M. (1994) Peritrophic matrix of the black fly *Simulium vittatum*: formation, structure, and analysis of its protein components. The Journal of Experimental Zoology, 268, 269–281. 8195743 10.1002/jez.1402680403

[mve12509-bib-0034] Reid, G.D.F. & Lehane, M.J. (1984) Peritrophic membrane formation in three temperate simuliids, *Simulium ornatum*, *S. equinum* and *S. lineatum*, with respect to the migration of onchocercal microfilariae. Annals of Tropical Medicine and Parasitology, 78, 527–539. 6524996 10.1080/00034983.1984.11811859

[mve12509-bib-0035] Rose, C., Casas‐Sánchez, A., Dyer, N.A. *et al*. (2020) *Trypanosoma brucei* colonizes the tsetse gut via an immature peritrophic matrix in the proventriculus. Nature Microbiology, 5, 909–916. 10.1038/s41564-020-0707-z. 32313202

[mve12509-bib-0036] Rovaris, K., Queiroz, P.M., Vasconcelos, K.d.F., Corpas, L.d.S., Silveira, B.M. & Freitas, D.Q. (2018) Segmentation methods for micro CT images: a comparative study using human bone samples. Brazilian Dental Journal, 29, 150–153. 10.1590/0103-6440201801385. 29898060

[mve12509-bib-0037] Sádlová, J. & Volf, P. (2009) Peritrophic matrix of *Phlebotomus duboscqi* and its kinetics during *Leishmania major* development. Cell Tissue Research, 337, 313–325. 10.1007/s00441-009-0802-1. 19471970 PMC2716444

[mve12509-bib-0038] Terra, W.R., Barroso, I.G., Dias, R.O. & Ferreira, C. (2019) Molecular physiology of insect midgut. Advances in Insect Physiology, 56, 117–163. 10.1016/bs.aiip.2019.01.004.

[mve12509-bib-0039] Trpis, M., Wergin, W.P. & Murphy, Ch, A. (2001) Development of *Onchocerca volvulus* (Filaroidea: Onchocercidae) in the West African black fly *Simulium yahense* (Diptera: Simuliidae) in Liberia. Journal of Parasitology, 87, 1265–1272. 10.1645/0022-3395(2001)087[1265:DOOVFO]2.0.CO;2 11780809

[mve12509-bib-0040] Vinkeles Melchers, N.V.S., Mollenkopf, S., Colebunders, R. *et al*. (2018) Burden of onchocerciasis‐associated epilepsy: first estimates and research priorities. Infectious Diseases of Poverty, 7, 101. 30253788 10.1186/s40249-018-0481-9 PMC6156959

